# The growth of a culture of evidence-based obstetrics in South Africa: a qualitative case study

**DOI:** 10.1186/1742-4755-8-5

**Published:** 2011-03-28

**Authors:** Karen Daniels, Simon Lewin

**Affiliations:** 1 Health Systems Research Unit, Medical Research Council, South Africa; 2 Faculty of Public Health and Policy, London School of Hygiene and Tropical Medicine, UK; 3 Norwegian Knowledge Centre for the Health Services, Oslo, Norway

## Abstract

**Background:**

While the past two decades have seen a shift towards evidence-based obstetrics and midwifery, the process through which a culture of evidence-based practice develops and is sustained within particular fields of clinical practice has not been well documented, particularly in LMICs (low- and middle-income countries). Forming part of a broader qualitative study of evidence-based policy making, this paper describes the development of a culture of evidence-based practice amongst maternal health policy makers and senior academic obstetricians in South Africa

**Methods:**

A qualitative case-study approach was used. This included a literature review, a policy document review, a timeline of key events and the collection and analysis of 15 interviews with policy makers and academic clinicians involved in these policy processes and sampled using a purposive approach. The data was analysed thematically.

**Results:**

The concept of evidence-based medicine became embedded in South African academic obstetrics at a very early stage in relation to the development of the concept internationally. The diffusion of this concept into local academic obstetrics was facilitated by contact and exchange between local academic obstetricians, opinion leaders in international research and structures promoting evidence-based practice. Furthermore the growing acceptance of the concept was stimulated locally through the use of existing professional networks and meetings to share ideas and the contribution of local researchers to building the evidence base for obstetrics both locally and internationally. As a testimony to the extent of the diffusion of evidence-based medicine, South Africa has strongly evidence-based policies for maternal health.

**Conclusion:**

This case study shows that the combined efforts of local and international researchers can create a culture of evidence-based medicine within one country. It also shows that doing so required time and perseverance from international researchers combined with a readiness by local researchers to receive and actively promote the practice.

## Research Evidence and Obstetrics

A shift has taken place in obstetrics over the past three decades [1,2]. This shift has seen a growth in the use of evidence in the fields of obstetrics and midwifery alongside the growth of evidence-based medicine more broadly [1,3-5]. There is also a growing awareness of the need to extend evidence-based obstetric practice and policy in low- and middle-income countries (LMICs) [6-12]. Several studies across Africa, Asia, the Middle East and Latin America have shown a lack of awareness amongst health workers of effective evidence-based obstetric interventions and a consequent failure to implement these interventions [7,9-12]. One of the key issues emerging from this literature is the poor access to information on effectiveness among health workers in LMICs [7,9-12]. Creating electronic and other platforms through which evidence-based health information in general [13,14], and information specific to obstetrics and gynaecology [6], can be accessed has been a key task of the World Health Organisation. Infrastructural barriers, particularly a lack of access to the internet (which is slow and expensive in many LMICs), have unfortunately hampered these attempts [15-18]. In an effort to bypass some of these barriers, the Reproductive Health Library (RHL) has been distributed as portable electronic files (mostly on compact discs) [6]. The RHL is aimed at low-income countries and contains mainly systematic reviews from the Cochrane Library, but also incorporates expert commentaries, guidance documents and implementation aids [6]. A randomised controlled trial across two country settings showed, however, that even after active dissemination and promotion of the RHL at hospital level, consistent or substantive changes to clinical practices were still not detected [6]. In other words, evidence-based practice had not increased as a consequence of the intervention tested in this trial setting. An important question, then, is why initiatives such as this are not successful and, following from this, how evidence-based obstetric practice can be facilitated in such settings?

Research on the process of knowledge translation has generated a considerable body of work on both the range of factors influencing the uptake of evidence into policy and practice [19,20] and the effects of initiatives to improve the uptake of evidence into policy and practice [6,21]. However the process through which a culture of evidence-based practice develops and is sustained within particular fields of clinical practice has not been well documented, particularly in LMICs. In a study of policy making for maternal health in South Africa we found that policies were strongly evidence-based [22]. This contrasts with the somewhat less convincing picture of a shift towards evidence-based obstetric practice presented above. This raises the question of how this positive outcome was achieved in this setting. In an attempt to draw lessons for future interventions, and for evidence-informed decision making in health more generally, this paper describes the development of a culture of evidence-based practice amongst maternal health policy makers and senior academic obstetricians in South Africa. While the factors associated with the translation of research evidence into policy in this setting are described in another paper [22], this paper focuses on the process through which evidence-based medicine became accepted. These studies form part of a larger programme of work on the uptake of evidence into policymaking in South Africa, Mozambique and Zimbabwe [23].

## Methods

The findings reported below tell the story of the development of a culture of evidence-based obstetrics in South Africa. These findings emerged from a broader qualitative case study which focused on policies regarding the management of eclampsia and severe pre-eclampsia, and particularly the use of magnesium sulphate in the management of these conditions [23]. The focus of the case study on pre-eclampsia and eclampsia reflects the important contribution of these conditions to maternal and infant morbidity and mortality in low-income countries [24-26]. In addition, strong evidence is available from randomised controlled trials (RCTs) of the effectiveness of magnesium sulphate for women with eclampsia and severe pre-eclampsia [27-31].

A qualitative case study approach was adopted in light of the complexity of the issues involved and in an attempt to understand the phenomena under investigation, as experienced by the actors involved in these [32,33]. Exploring actors' own accounts of their experiences enhances understanding of the *processes*, rather than simply the *outcomes*, of research utilisation. Throughout we attempted to adopt a reflexive stance by maintaining an awareness of our influence over the research process and outcomes [34].

### Data collection

The case study is built on a triangulation [35] of three data collection methods: a document review, the development of a timeline and fifteen key informant interviews with local researchers and policy makers. All of these methods have been used in previous studies of the utilisation of research information in health policy [36]. Although this paper does not focus on policy development, each of these methods contributed to our understanding of the growth of a culture of evidence-based obstetrics.

#### Document review

Copies of all contemporary national policies and guidelines were obtained from the National Department of Health. These were reviewed with the aim of establishing the extent to which research information had been implicitly and explicitly used [37]. This was done through checking the references of each policy and the extent to which the use of research information was mentioned. Whether or not the policy recommended magnesium sulphate for the treatment of eclampsia and pre-eclampsia was also noted.

#### Timeline

A timeline of key events in the development of policy and practice for the management of eclampsia and pre-eclampsia in South Africa was constructed iteratively. At each interview respondents were asked to comment on or add to the timeline. The timeline was also shared with colleagues working in the field. Relevant bibliographical and conference databases, as well as the websites of organisations such as the National Department of Health in South Africa and the Cochrane Collaboration, were also searched for information.

#### Interviews

Between 2004 and 2005 individual qualitative interviews were conducted by KD and SL with fifteen local researchers based in tertiary units for obstetric care and midwifery and government officials (past and present). These interviews were structured around a flexible interview schedule designed to address all facets of the research question. The respondents were identified using a combination of purposive sampling (through which respondents were identified prospectively by the research team) and 'snowballing' (through which respondents were identified during the process of the research) [38]. In sampling, attention was given to fair dealing and seeking out negative cases [34]. Sampling was stopped once we had reached data saturation and once we were also assured that we had reached all relevant respondents. The interviews were audio-recorded and then transcribed verbatim. The analysis was conducted using these verbatim transcripts.

Our respondents had all been trained in either midwifery (4) or obstetrics and gynaecology (11). They were all either active in the policy and guideline development process or were very knowledgeable about this area. Four respondents were currently or formally employed by the National Department of Health. One respondent was a practising midwife and nursing tutor. The other eight were academic researchers (professors and associate professors). One respondent had held a senior position both in the national department and as an academic researcher.

### Analysis

An inductive thematic content analysis was conducted [39]. The process was lead by KD, supervised by SL and internally validated by the wider research team. After immersion in the transcripts, these were coded for both latent and manifest themes [40]. Data extracts assigned to each code were cut and pasted into a Word document, which was shared and checked between KD and SL. Out of this process a narrative account of the data was written up as the key research findings, some of which are presented here. The findings are illustrated further by data extracts selected on the basis of being representative and/or interesting illustrations of each of the themes [41].

When using an inductive approach to data coding, the researcher anticipates that themes and issues will emerge from the data set rather than applying a predefined set of codes to the data. Out of our initial analysis of the data on knowledge translation for policies on the management of pre-eclampsia/eclampsia, the growth of a culture of evidence-based medicine emerged as a major theme. Thus we felt that this important component of the process needed discussion in its own right and not just as a part of our understanding of knowledge translation. This paper therefore focuses on what we have learnt about the growth of a culture of evidence-based medicine in obstetrics through our broader study of knowledge translation.

## Ethical issues

The main study received approval from ethics committees at the Medical Research Council of South Africa, the London School of Hygiene and Tropical Medicine and the World Health Organisation. This sub-study also received approval from the ethics committee chairperson at the University of Cape Town. All participants in the study signed a consent form after being given a written study information sheet and a verbal explanation of the consenting process.

## Findings

The concept of evidence-based medicine became embedded in South African academic obstetrics at a very early stage in relation to the development of the concept internationally. The diffusion of this concept into local academic obstetrics was facilitated by contact and exchange between local academic obstetricians, opinion leaders in international research who promoted evidence-based practice and structures promoting evidence-based practice, such as the Cochrane Collaboration and the earlier Oxford Database of Perinatal Trials. Furthermore the growing acceptance of the concept was stimulated locally through the use of existing professional networks and meetings to share ideas and the contribution of local researchers to building the evidence base for obstetrics both locally and internationally. The existence of strongly evidence-based policies for maternal health in South Africa is testimony to the extent to which evidence-based medicine has been diffused. Below we describe how this came about.

### The growth of evidence-based obstetrics internationally and in South Africa

#### International growth

One cannot explore the growth of evidence-based obstetrics in South Africa without considering the concurrent developments internationally. The literature describes well the determined attempts to make the science and practice of obstetrics internationally more evidence-based, and the subsequent growth of evidence-based obstetrics globally [1,2]. The late 1970s to early 1980s saw the development of the Cochrane Collaboration with involvement of obstetricians, midwives and others in improving, and systematising, the evidence base for their professions [2,42]. Obstetrics shifted from the days when it was criticised for being the least evidence-based medical speciality (1979), to being a forerunner in the Cochrane Collaboration, with the Pregnancy and Childbirth Group being the first Cochrane review group to be registered in 1992 [42].

#### Contact and Exchange

Because of the international academic boycott in response to apartheid, many international researchers were very concerned about accepting invitations to visit South Africa during the 1970s and 1980s. South African researchers, however, spent time at international centres during this time. Thus, despite the isolation of South African academic institutions, links between South African researchers and the Oxford Database and its leaders began in the 1980s [43]. Later these links prompted visits from leading international researchers. After wide consultation, Iain Chalmers visited South Africa in 1989. His decision to visit was influenced by the fact that the researchers he was to meet were concerned with improving obstetric care for disadvantaged groups. Other leading researchers in evidence-based obstetrics (including Murray Enkin, and Lelia Duley) also presented at the conferences of the Perinatal Association of South Africa [43,44]:

"We actually invited Murray Enkin, who was attached to the Oxford Database. And subsequent to that Iain Chalmers, who's the editor, was invited to attend. So we were sort of, I think from the word go, when the Oxford Database became available for use, we were part of it, we were aware of it, we were using it, and I think quite a few South Africans became involved on their editorial board and as editors or reviewers, or whatever." (Resp 1 pp. 2-3)

South African researchers who had spent time during their sabbaticals and fellowships working at the National Perinatal Epidemiology Unit in Oxford formed part of our respondent group. At least two of them are particularly influential within the local obstetric fraternity and were part of the core group of researchers who assisted in writing the current policies and guidelines for maternal health nationally and in their local institutions. All of them suggest that their exposure while working at this Unit in Oxford was important in shaping their thinking around research and evidence:

"And if you look at the proceedings of the first couple of conferences, most of the studies were epidemiological studies, and then you'll see, I think from the early 80s onwards, the move towards more and more randomized trials and systematic reviews were being presented. And I think it was really the influence of the Oxford Database of Perinatal Trials which got us all thinking in that way." (Resp 3 pp. 8-9)

Importantly, it was through these links that the Eclampsia Trial (published in 1995) included centres in Harare, Durban, Johannesburg and Pretoria, all lead by local researchers [30]. Through this collaboration, researchers based in LMICs were able to contribute to this international landmark trial on the management of eclampsia. This trial had major implications for poor countries because it provided evidence that a low cost drug was more effective than alternatives for the management of a life-threatening complication of a condition that continues to contribute to a very substantial proportion of maternal mortality in LMICs [26,45,46].

#### A platform from which to share ideas and build networks: local conferences

The attempts at an international level to promote evidence-based medicine, and the increased availability of evidence from trials through the Oxford Database of Perinatal Trials and later the Cochrane Collaboration, was met by eagerness within the South African obstetric fraternity. Alongside these international developments, locally organised professional conferences showed an openness to new ideas around evidence-based medicine. For example, in 1982 the Perinatal Priorities Association of South Africa began hosting an annual conference attended by local, regional and international health professionals [43,44]. As their conference database shows, these annual meetings offered a platform for publicising both locally generated and international research [43,44]. The data suggests that exposure to the idea of 'evidence' at this conference, as well as that hosted by the South African Society of Obstetrics and Gynaecology, was an important factor in promoting a culture of evidence-based medicine:

"But I think that one very important aspect is, sort of, the culture of research within a department, and the second one is exposure to events, like for instance, the Priorities in Perinatal Care Conference where you gain knowledge and where you interact, not only with other departments in the country but also with overseas experts. That was very, very valuable...." (Resp 1 p. 3)

Regular attendance at these conferences allowed for the emergence of a closely linked national network of researchers who promoted evidence-based medicine in their own academic departments and nationally. One respondent remarked that as a consequence of his obstetrics training, evidence-based medicine was "the truth I knew - that's how I was cultured" (DoH 3). Within academic departments the data suggest that this idea was promoted through teaching, journal clubs and also extensively through involvement in research:

"...so evidence, that sort of thing was really, ja [yes], grasped with both hands. I think a lot of our research is clinical. So trials are our - if you want to do research - is our bread and butter. We are not going to really be able to do much on the biochemical research because of [lack of] resources and so on. So our research and our strengths are in clinical trials.... I don't think there's any institution out there, O & G [obstetric and gynaecological] academic institution, which doesn't use Cochrane extensively." (Resp 7 p. 21)

#### Local contributions to the international evidence base

The international trend or shift in consciousness around evidence was replicated within academic obstetric practice in South Africa. South African academics were not only the passive recipients of international evidence. They were also reviewing it in relation to health conditions being treated locally - for example, in 1980 a researcher at the University of Natal published the results of a survey of hypertension in pregnancy as experienced at the hospital in which he worked as well as a review of the management of hypertension in pregnancy [47,48]. Similarly, South African academic obstetricians contributed to international evidence by conducting research in their settings, including RCTs. For example, in the late 1980s a senior academic obstetrician at the University of Cape Town conducted a trial comparing phenytoin sodium and magnesium sulphate in the treatment of eclampsia [49].

### Evidence-based policies: an outcome of this culture

1994 saw the election of the first democratic government in South Africa. This shift brought about changes in the national management of maternal health including new values and a new policy making staff [22]. Amongst this staff were people linked closely to local obstetrics and gynaecology academic networks [22]. Through their appointment, as well as strong lobbying by the local obstetrics and gynaecology network and a general climate of policy change, new policies for maternal health care were developed [22,50,51]. These policies were strongly evidence-based [22,52-54]. The high value attributed to evidence in the policy making process is clear in this data extract from one of the early policy developers:

"But I mean, in the world we live today, it is the world of evidence-based practice, evidence-based medicine. So one had to be careful about... cognizant of the fact that one had to use the best available evidence. Because I mean, this would be national... it would be a reflection of us as a country." (DoH 3)

It was very clear from this set of interviews that respondents' conceptualisation of evidence reflected the same understanding of the term promoted by proponents of evidence-based medicine internationally [55]. The interviews suggest that respondents considered the findings of systematic reviews and RCTs as highest on the hierarchy of research evidence regarding the effects of clinical interventions. The following data extract reflects how most of the respondents related to the concept of evidence:

"No, it [the Perinatal Education Programme manual] was purely evidence-based. We really tried to be as scientific as possible... the Oxford Database [of Perinatal Trials] was used very extensively, and subsequent to that the Cochrane database. So we tried to stick as close as possible to evidence-based medicine and not sort of traditional ways and means of dealing with things, but really to make it, have it scientifically founded." (Resp 1 p. 2)

What this data extract reveals is a sense of evidence-based practice and decision making as being more scientific and therefore 'better' or more sound than the traditional practice of medicine. It carries with it a sense of being modern rather than old fashioned. In another data extract, a respondent links evidence-based medicine with best practice, even suggesting that a sense of prestige surrounds the practice of evidence-based medicine:

"At that stage [the 1970s], I was a member of [an international obstetrics] society and attended all their congresses, and I was director of [a] perinatal mortality research unit... So of course, you wanted to do the best practice. You wanted to use the best protocols. You wanted to do evidence-based medicine. And our connections with the Cochrane library also started very long ago when it still used to be the Oxford Database of Perinatal Trials, and Iain Chalmers, who once gave a lecture in South Africa. [1989], I once visited him in London. So we became aware of randomised controlled trials very, very early.... He quoted me in his talk and saying that we were one of the first South Africans to do randomised controlled trials" (Resp 6 p. 5)

The conceptualisation of 'evidence' as being based on the findings of RCTs and systematic reviews, and as a key element of scientific best practice, was noted from the start of interviewing. We also found that this conceptualisation was present and influential in the writing of national policies and guidelines for maternal care. Following the democratic elections of 1994, the following guidelines for maternal care were produced by the new administration:

• *Guidelines for Maternity Care in South Africa: A manual for clinics, community health centres and district hospitals*, (2000 & 2002)[53]

• *Saving Mothers: Policy and Management Guidelines for Common Causes of Maternal Deaths*, (2001) [54]

• *Saving Mothers: Second Report on Confidential Enquiries into Maternal Deaths in South Africa, 1999-2001*, (2002)[52]

Review of these policy documents showed very concrete and explicit examples of research utilisation in policy making and guideline development. For example, both of the editions of the Guidelines for Maternity Care carry in their introduction a statement reading: "The guidelines are based on the best available evidence from published research, modified where necessary to suit local conditions". We also observed a strengthening in the use of research evidence in policy documents over time. While the early policies merely informed the readers that evidence was used, the later documents cited which evidence was used for specific recommendations, and also listed the grade or strength of this evidence. Thus for example in its recommendation for eclampsia treatment, the Saving Mothers Policy and Management Guidelines state: "Magnesium sulphate is the best drug to arrest and prevent further convulsions Grade A evidence. (The Eclampsia Collaborative Group. Lancet 1995; 345:1455-63)".

### Some local scepticism

Despite extensive attempts to identify differing views, our respondents included only one person who was not wholly supportive of evidence-based medicine. This person (Resp 8) felt that the use of evidence was important and to this end he taught the skills of evidence-based medicine to his obstetric registrars (trainees). However, he also felt that there was a lack of critical appraisal in the use of evidence. He felt that sometimes proponents of evidence-based medicine were more interested in the methods than in the relevance of question. This respondent added that patients needed to be treated individually, calling for the use of clinical judgement, not only adherence to the evidence. Furthermore he felt that the conduct of trials had become an industry. This debate regarding the application of research evidence to policy questions and individual clinical cases is long standing. Alongside the growing strength of evidence-based medicine, there is also wide acceptance amongst its proponents that randomised controlled trials need to be combined with other information, such as the values of preferences of service users and other stakeholders, to address complex clinical and health policy questions [2,56].

## Discussion

The success with which a culture of evidence-based medicine spread within senior obstetrics networks in South Africa stands in sharp contrast to the literature describing a lack of access to and awareness of evidence in many LMICs [7,9-18]. What lessons then can we draw from this study of this process in South Africa that may assist others hoping to achieve similar outcomes?

The first key lesson is that this process, as well as the growth of evidence-based obstetrics internationally, was a consequence of considerable time and effort on the part of a large number of highly motivated and skilled individuals and networks, who acted as champions in advocating this approach. The presence, in the South African context, of a fairly large number of senior, locally trained academic obstetricians who had worked in the field over long periods of time, thereby creating a 'critical mass' of people with shared views, may have further contributed to the development of a culture of evidence-based medicine. This facilitator may not be present in a number of other low- and middle-income countries, where the number of senior obstetricians may be very small.

It is also worth noting that the widespread culture of evidence-based medicine amongst our respondents took twenty years to cultivate. Given the length of time that it took for evidence-based medicine to diffuse into obstetric practice internationally, this is hardly surprising. Reflecting on the international process, Murray Enkin eloquently states:

"This pioneering approach received academic accolades, but seemed to have little influence on obstetrical practice. It took what seemed to us to be ages before the profession and the public began to appreciate how effective randomized trials could be as a way to choose between alternative forms of care. We should have been more patient; shifts in paradigms do not occur quickly."[2] (p266)

A second key lesson is that the idea of 'evidence', and particularly evidence as constituting the findings of RCTs and systematic reviews, seems to have spread through personal contact and advocacy. Recent efforts such as HINARI (a programme for access to published health research in LMICs) and the Reproductive Health Library rely on health professionals coming into contact with the evidence electronically. Unfortunately both of these interventions have met with challenges [6,18]. HINARI reportedly did not include access to key medical journals while training in the use of the Reproductive Health Library did not lead to changes in clinical practice [6,18]. What we see in the example presented in this paper is the spread of the concept of evidence-based medicine long before the electronic information revolution. This concept was transported person-to-person. It spread through interpersonal contact - including sabbaticals, conferences and academic teaching. This suggests that as much as the information revolution holds great potential, one-to-one 'talk time' may be equally important. This is echoed in systematic reviews of research utilisation by policy makers where interpersonal interaction between researchers and policy makers was found to promote the uptake of evidence [19,20]. A valid counter argument would be that such interpersonal contact is expensive. However, targeting only opinion leaders for exchange efforts may reduce such costs. Also encouraging visits from leaders in the field to LMICs may be well worth the effort.

Thirdly, the idea of evidence was spread despite the academic isolation of South Africa as a consequence of apartheid. This seems to have been due in part to personal determination on the part of both South African researchers and their international counterparts to ensure that evidence-based interventions that could improve maternal health in disadvantaged communities were translated into policy and practice.

In summary then, the growth of a culture of evidence-based medicine amongst obstetricians in South Africa took patience, interpersonal contact and personal determination on the part of a number of key champions. Furthermore, adopting an evidence-based approach was associated with being 'modern' or keeping abreast of the times scientifically. This attitude permeated the policymaking process where at least some key policy developers felt that it was important for the country that its policies be seen to be evidence-based. Of course, evidence-informed maternal health policies are only part of the complex package needed to improve maternal health in settings such as South Africa. For example, despite the robustness of evidence for the provision of continuous support for women during labour [57], researchers in South Africa have struggled to implement this intervention widely [58]. They conclude that "more consideration should be given to seeking political commitment from health authorities and senior staff within hospitals, and public awareness campaigns to promote greater sustainability" (p [58].

Constraints such as over extended staff and health resource shortages also need to be addressed [2] and these relate to a wider body of evidence on the effects of different health systems arrangements [59]. Furthermore McCourt (2005) points out that randomised controlled trials (the gold standard for evidence-based medicine) cannot in themselves describe the complexity of the context in which knowledge is generated [56]. Thus it may be worth exploring both the context in which contemporary evidence is created and the context into which it is being delivered and received. Those attempting to enhance knowledge translation need to consider how global evidence can be applied within a specific context and how that context could be made more receptive. Efforts to develop tools to support this are currently underway [60,61].

This paper has two key limitations. Firstly we did not set out in this study to explore the development of a culture of evidence-based medicine. Rather, we discovered its existence in the process of understanding the relationship between evidence and policy. This means that we may not have explored the topic to its full extent. A second limitation is that we only interviewed senior members of the obstetrics and gynaecology fraternity since they were key figures in policy development. Our focus on this 'policy elite' means that we have little direct data on whether this culture of evidence-based medicine permeated through the maternal health domain more broadly. This is a common constraint of policy analysis case studies [62].

## Conclusions

This case study from South Africa shows that it is possible to create a culture of evidence-based medicine that facilitates and supports evidence-based practice by obstetricians. Enabling this culture in this setting, however, took time, perseverance and effort, both from local researchers and their colleagues internationally. Importantly, because local researchers were already open to the concept of evidence-based practice and were organising themselves to promote this, they proved to be fertile ground for initiatives brought by international proponents of this approach to health care delivery.

## Competing interests

The authors declare that they have no competing interests.

## Authors' contributions

KD conducted the research and wrote the paper supervised by SL who was the principal investigator for the study in South Africa. They were supported actively in the process by the Practihc Policy Group. All authors read and approved the final manuscript.
